# Randomized Phase II Study of Brentuximab‐Vedotin With High‐Dose Chemotherapy in CD30 Positive Lymphoma

**DOI:** 10.1002/hon.70143

**Published:** 2025-10-21

**Authors:** Christian Rausch, Ulrike Bacher, Manuela Rabaglio, Corinne Vorburger, Anke Klingenberg, Yara Banz, Michael Daskalakis, Thomas Pabst

**Affiliations:** ^1^ Department of Medicine III LMU University Hospital Munich Germany; ^2^ Department of Hematology and Central Hematological Laboratory Inselspital University Hospital Bern University of Bern Bern Switzerland; ^3^ Department of Medical Oncology Inselspital University Hospital Bern University of Bern Bern Switzerland; ^4^ Institute of Tissue Medicine and Pathology University of Bern Bern Switzerland

**Keywords:** autologous stem‐cell transplantation, brentuximab‐vedotin, hodgkin lymphoma, peripheral T‐cell lymphoma, phase II study

## Abstract

Patients with Hodgkin lymphoma (HL) or peripheral T‐cell lymphoma (PTCL) who relapse after high‐dose chemotherapy (HDCT) have a dismal prognosis. Brentuximab‐vedotin (BV) is a CD30‐targeting antibody‐drug‐conjugate (ADC) used in first‐line‐, salvage‐, and maintenance‐therapy of HL, as well as first‐line‐ and salvage‐therapy of PTCL. In phase I of this trial, we could show that BV can safely be added to BeEAM‐HDCT (bendamustine, etoposide, cytarabine and melphalan). Here, we report the randomized phase II part of the trial comparing BV‐BeEAM to BeEAM alone (ClinicalTrials.gov: NCT03187210). Primary endpoint was 1‐year disease‐free survival. Inclusion of 42 patients was planned but the study was terminated early, after a futility analysis showed lack of benefit. Twenty‐five patients (HL: 11, PTCL: 14) who were planned to undergo HDCT were included. Median age was 60 years. Patients had a median of two prior therapies, and 11 were previously exposed to BV. Patients in the standard‐arm had higher disease stage and (PTCL only) higher IPI. Duration of hospitalization, recovery of neutrophils/platelets, and infections were not significantly different between arms. No treatment related death occurred. However, two patients in the BV‐arm developed grade 3 pneumonitis. After 22 months median follow‐up, overall response‐rate, complete remission‐rate, disease‐free survival and overall survival did not differ between groups. Pre‐planned subgroup‐analyses (HL‐only, PTCL‐only, only those achieving CR) did not show benefit in any subgroup. In conclusion, adding BV to BeEAM does not improve outcomes after HDCT, and may increase pulmonary toxicity. Frequent prior exposure to BV may have limited the potential benefit of the combination.

## 1 Introduction

CD30+ lymphomas comprise Hodgkin lymphoma (HL) and several peripheral T‐cell lymphomas (PTCL) including angioimmunoblastic T‐cell lymphomas (AITL), anaplastic large‐cell T‐cell lymphomas (ALCL), PTCL‐NOS, and numerous rare entities. For all these entities, high‐dose chemotherapy (HDCT) with autologous stem cell transplantation (ASCT) is part of the therapeutic algorithm. In PTCL, ASCT in first remission is the preferred consolidation therapy, achieving up to 49% 5 year‐PFS in AITL [1]. Most HL can be cured using combination chemotherapies, but in relapsed or refractory (r/r) HL, HDCT with ASCT cures around 50 percent of patients [2], with maintenance therapy with the CD30‐targeting antibody‐drug‐conjugate (ADC) brentuximab‐vedotin (BV) improving outcomes even further for high‐risk disease (5 years‐PFS 59% in the AETHERA trial) [3, 4]. At many centers, the preferred HDCT regimen before ASCT is BeEAM (bendamustine, etoposide, cytarabine and melphalan), as its efficacy is similar to BEAM (carmustine, etoposide, cytarabine and melphalan), but it avoids carmustine‐related pulmonary toxicities, and its commercial availability is more reliable [5, 6, 7, 8, 9, 10]. The prognosis of patients relapsing after ASCT is poor, and response rates decline with every further line of therapy. As reaching a deep and durable remission after HDCT is paramount, we explored the possibility to improve responses to HDCT by adding BV to BeEAM‐HDCT.

BV combines an anti‐CD30 antibody with monomethylauristatin‐E, an antimitotic agent. It has initially shown promising response rates of up to 75% in r/r HL, with a minority of patients acheiving long‐lasting remissions [11, 12, 13, 14, 15]. Since then, BV has become standard of care in first‐line therapy of advanced HL as part of the BrECADD‐regimen [16]. Additionally, BV together with different chemotherapies (AVD, bendamustine, DHAP, ESHAP, ICE), checkpoint‐inhibitors (ipilimumab/nivolumab, pembrolizumab), or the BTK‐inhibitor ibrutinib has shown efficacy in the salvage therapy of r/r cHL [17, 18, 19, 20, 21, 22, 23, 24, 25]. In addition, multiple real‐world analyses confirm the benefit of BV‐maintenance after ASCT [26, 27, 28].

In CD30+ PTCL first‐line treatment, a randomized phase III trial showed a survival advantage of CHP (cyclophosphamide, doxorubicin prednisone) and BV over CHOP [29, 30]. In relapsed ALCL, BV‐monotherapy induced complete remission (CR) in 57% of patients in a phase II trial [31]. Long‐term responses have been observed in a subset of patients [32].

In light of these promising results we considered the addition of BV to BeEAM to induce deeper remissions in CD30+ lymphomas prior to ASCT. In a phase I trial we added one dose of BV (0.9–1.8 mg/kg b.w.) to BeEAM. In a heavily pretreated population, across all BV dose‐levels a promising CR‐rate of 75% was reached [33]. In addition, even at the highest dose level, no toxicities beyond what would be expected with BeEAM alone were apparent. We hypothesized that even better results could be obtained when using only the target‐dose of 1.8 mg/kg b.w. in conjunction with HDCT. This randomized phase II study compares HDCT alone or together with one dose of BV.

## 2 Patients and Methods

### 2.1 Study Design

This phase II trial is a randomized, single‐center, open‐label trial. Key inclusion criteria included the diagnosis of CD30 positive lymphoma in first or second remission or a second chemo‐sensitive relapse in patients considered fit to undergo HDCT/ASCT. Prior use of BV and BV‐maintenance were permitted. Local pathology was confirmed by central reference pathology. Further inclusion criteria comprised age 18–75 years, adequate liver and kidney function (creatinine < 2.0 mg/dL, bilirubin < 1.5x ULN, transaminases < 3x ULN) and sufficient marrow function (Hb ≥ 8 g/dL, Plt ≥ 75.000/μL). Key exclusion criteria were concurrent malignant disease, uncontrolled cardiovascular or infectious disease, and pregnancy or lactation. Full inclusion and exclusion criteria are listed in Supporting Information S1: Table 1. All patients gave written informed consent. The study protocol and related amendments were approved by the local ethics committee (ethics committee Bern, #2016‐01351). The study was conducted in accordance with the declaration of Helsinki and the International Conference on Harmonization Guideline for Good Clinical Practice, and it was registered in relevant databases (EudraCT‐number: 2015‐004266‐28; ClinicalTrials.gov number: NCT03187210).

### 2.2 Study Intervention

After screening, patients were randomized in a 1:1 fashion. Inclusion of 42 evaluable patients including 6 patients treated at the same BV‐dose in the preceding phase I trial was planned to achieve a power of 80% for detection of a 20%‐difference of one‐year DFS between arms [33]. However, a futility analysis requested by the sponsor (University Hospital Bern) after randomization of 19 patients led to early discontinuation of the trial.

Patients in the standard arm were treated with BeEAM‐HDCT supported by subsequent ASCT. The regimen consisted of bendamustine 200 milligram per square meter body surface area per day (mg/m2/day) given as a single infusion in 500 ml NaCl 0.9% over 120 min on days −7 and −6 supported by volume supplementation with 2000 ml NaCl 0.9%. Etoposide 200 mg/m2/day, and cytarabine 400 mg/m2/day were infused in 500 ml NaCl 0.9% over 30 min each on days −5 through −2. Melphalan 140 mg/m2, given as a single infusion in 500 ml NaCl 0.9% over 60 min, was given on day −1, also supported with supplementation of 2000 ml NaCl 0.9%. A minimum of 2.0 × 10e6 autologous stem cells/kg bodyweight (BW) were given for ASCT at day 0.

In addition to the standard BeEAM regimen, patients in the experimental arm received 1.8 mg/kg b.w. BV suspended in sterile water (5 mg/ml) and given as a single intravenous infusion over 30 min on day −8.

We administered antiemetic prophylaxis according to local standards. G‐CSF was given s.c. in all patients starting at day +6 and continued until day +12 after ASCT. All patients received valaciclovir (500 mg twice daily) for 3 months after ASCT, and trimethoprim/sulfamethoxazole (160 mg/800 mg 3 days a week) for 3 weeks after ASCT, as well as fluconazole (400 mg once weekly) until 3 weeks after ASCT. Patients received platelet or red blood cell transfusions as needed.

Patients were hospitalized before application of BV, and discharged after adequate physical and hematologic recovery after ASCT. BV was provided by Takeda Pharmaceuticals while commercially available products were used for BeEAM. Patients with cHL were planned to undergo BV maintenance after ASCT.

### 2.3 Study Assessments and Definitions

Before HDCT, response was assessed by PET‐CT (*n* = 13) or conventional CT (*n* = 12). Patients were observed for 12 months after ASCT and were followed thereafter for survival on an annual basis until study discontinuation. Causes for discontinuation were withdrawal of consent, lack of compliance, relevant protocol violation, unacceptable toxicity or death and loss to follow‐up. Response assessment was performed by the investigators in accordance to the International Harmonization Project Group 2007 Revised Response Criteria [34]. The data cut‐off was Dec 31, 2024.

Staging was performed according to the Ann‐Arbor criteria. For NHL, the International Prognostic Index (IPI) was calculated at diagnosis. Standard histopathology, immunohistochemistry, and molecular studies were performed. During the entire study, all adverse events (AEs) (except alopecia and nausea/vomiting/loss of appetite, which were considered expected side‐effects of HDCT) and all serious adverse events (SAEs) were protocoled, fully investigated, and assessed using the NCI CTCAE version 4.03.

The primary endpoint of this trial was disease‐free‐survival (DFS) at 12 months. Secondary efficacy outcomes included overall response rate (ORR), progression free survival (PFS), and overall survival (OS). Secondary safety outcomes included time to hematologic recovery as well as all adverse events (AEs) and severe adverse events (SAEs) observed on trial.

### 2.4 Statistical Analyses

We estimated that 30% of patients would relapse within 1 year after BeEAM‐HDCT. Thus, 42 patients would provide 80% power to detect an improvement margin of 20% with the use of a log‐rank test at a one‐sided significance level of 0.02.

Quantitative variables are summarized using median and range, while categorical variables are presented as the absolute frequency and percentage of patients in each category. Associations between treatment arm and other variables were analyzed using Fisher's exact test for binary and the Wilcoxon rank‐sum test for ordinal and continuous variables. AEs are presented by type and grade using frequency and percentage of within‐patient worst grades. In addition, grade ≥ 3 AEs are summarized separately. Time‐to‐event endpoints were analyzed using the Kaplan‐Meier method. Survival between groups was compared using the log‐rank‐test. Prespecified stratified analyses were performed, with type of lymphoma (HL vs. TCL) and remission status at ASCT (CR vs. no CR) serving as stratification factors. All calculations were performed on a strict intention‐to‐treat basis. *p* values < 0.05 were considered significant. Data were analyzed using R version 4.4.1 (R foundation for Statistical Computing, Vienna, Austria). Undermind.ai was used to aid literature search, with any suggestions undergoing careful review by the authors.

## 3 Results

### 3.1 Patient Cohort

**FIGURE 1:**
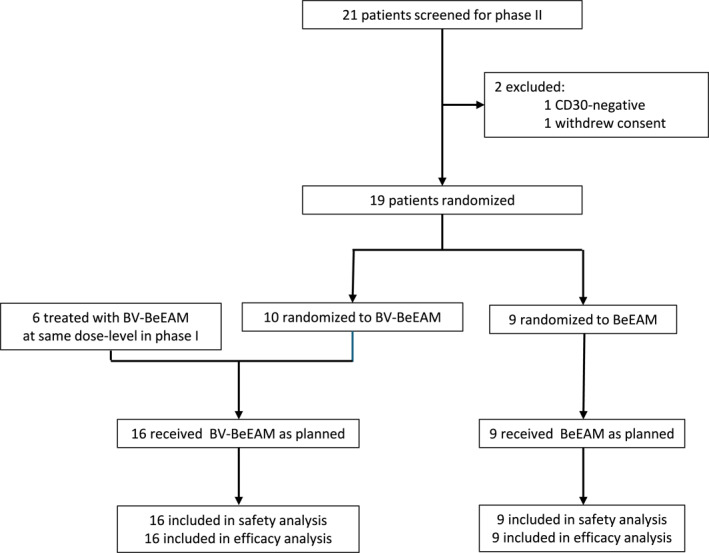
Consort diagram detailing screening, randomization, and treatment of patients. 21 patients were screened for the randomized phase II part of this trial, of which 19 could be included. Per protocol, the experimental arm was augmented by the six patients treated at the same BV dose‐level in phase I (single‐arm, dose‐finding study) of this trial.

Of 21 screened patients, nine patients were randomized to BeEAM and 10 to BV‐BeEAM between 07.05.2021 and 24.08.2023 (Figure 1). One patient was CD30‐negative in reference pathology and one withdrew consent before they could be randomized. No further patients were screened, as a futility analysis at the request of the sponsor based on findings of the data monitoring committee recommended termination of the study. In addition, six patients treated in the phase I part of the trial were included in the experimental arm, for a total cohort of 25 patients. Characteristics of the cohort are detailed in Table 1, and stratified cohort characteristics for HL (*n* = 11) and PTCL (*n* = 14) are shown in Supporting Information S1: Tables 2–3. The PTCL comprised AITL (*n* = 5), ALCL (*n* = 2), EATL (*n* = 1), nTFHL‐NOS (*n* = 2) and PTCL‐NOS (*n* = 4). Median age for all patients was 60 years (range: 19–72). There was a male predominance (72%) which was even more pronounced in the HL subcohort (82%). Most patients had advanced disease with 21/25 presenting with stage III or IV lymphoma. Patients in the standard arm were more likely to have a higher disease stage (*p* = 0.01), In addition, PTCL‐patients in the standard arm were more likely to have a higher IPI (*p* = 0.02) and higher CD30‐expression (*p* = 0.04). Patients had a median of 2 lines of prior therapy, with 44% having previously been exposed to BV. The most frequent prior therapies for HL were DHAP, BEACOPP, and ABVD. In PTCL, BV‐CHP, CHOP, and CHOEP were most frequently used. All prior therapies are listed in Supporting Information S1: Tables 2–3.

**TABLE 1:** Baseline characteristics.

Demographic characteristics	All	BeEAM	BV‐BeEAM	*p*‐value
*n*	25	9	16	—
Age at ASCT, y, median (range)	60 (19–72)	60 (39–71)	60 (19–72)	1
Gender, female, *n* (%)	7 (28%)	2 (22%)	5 (31%)	1
HL, *n* (%)^a^	11 (44%)	3 (33%)	8 (50%)	
TCL, *n* (%)^a^	14 (56%)	6 (67%)	8 (50%)	
CD30+ lymphocytes at diagnosis, %, median (range)	90 (5–100)	100 (40–100)	90 (5–100)	0.2
Stage (ann‐arbor), *n* (%)				
I	1 (4%)	0 (0%)	1 (6%)	**0.01**
II	3 (12%)	2 (22%)	1 (6%)	
III	7 (28%)	1 (11%)	6 (38%)	
IV	14 (56%)	6 (67%)	8 (50%)	
IPI, *n* (%) (NHL only)				
0	0 (0%)	0 (0%)	0 (0%)	**0.02**
1	3 (21%)	1 (7%)	2 (14%)	
2	5 (36%)	2 (14%)	3 (21%)	
3	5 (36%)	2 (14%)	3 (21%)	
4	0 (0%)	0 (0%)	0 (0%)	
5	1 (7%)	1 (7%)	0 (0%)	
ECOG‐PS, *n* (%)				
0	23 (92%)	8 (89%)	15 (94%)	0.1
1	2 (8%)	1 (11%)	1 (6%)	
Extranodal involvement, *n* (%)^b^	13 (52%)	5 (56%)	8 (50%)	1
Previous therapies				
Prior lines of chemotherapy, median, (range)^c^	2 (1–4)	2 (1–3)	2 (1–4)	0.3
Prior radiotherapy, *n* (%)	3 (12%)	1 (11%)	2 (13%)	1
Prior exposure to BV	11 (44%)	5 (56%)	6 (38%)	0.4
Time from Dx to ASCT, months, median (range)	11 (6–199)	11 (6–199)	11 (6–91)	0.8
Remission status before ASCT, *n* (%)				
CR	17 (68%)	5 (56%)	12 (75%)	0.4
PR	6 (24%)	4 (44%)	2 (13%)	
SD	1 (4%)	0 (0%)	1 (6%)	
PD	1 (4%)	0 (0%)	1 (6%)	

*Note:* Bold formatting indicates *p* < 0.05.

Abbreviations: ASCT, autologous stem cell transplant; BV, Bentuximab‐Vedotin; CR, complete response; ECOG‐PS, eastern cooperative group performance status; HL, Hodgkin Lymphoma; IPI, international prognostic index; PD, progressive disease; PR, partial response; SD, stable disease.

^a^ Histologic subtypes are given in Supporting Information S1: Tables S1 and S2.

^b^ Locations are given in Supporting Information S1: Tables S1 and S2.

^c^ Regimens are given in Supporting Information S1: Tables S1 and S2.

### 3.2 Safety and Tolerability

All randomized patients received therapy as planned. Excluding expected AEs outlined above and cytopenias which we report separately, 15/25 patients had at least one any‐grade AE, with all 15 also having at least one grade 3–4 AE. There was no treatment‐related mortality.

Non‐cytopenia adverse events are listed in Table 2. Generally, the toxicities observed in the experimental arm were comparable to those seen with standard BeEAM and ASCT. The rate of infections in the experimental arm was 63% (10 of 16 patients) and 44% (4 of 9 patients) in the standard arm (*p* = 0.4), with all reported infectious AEs being grade 3–4 in both arms. Peripheral neuropathy was not increased in the BV‐containing arm. Of note, two cases of pneumonitis occurred in the experimental arm. Both were successfully treated with steroids and resolved completely.

**TABLE 2:** Adverse events, *n* (%).

	All			Grade 3–4		
	All	BeEAM	BV‐BeEAM	All	BeEAM	BV‐BeEAM
Infectious/Inflammatory						
Bladder infection	1 (4)	0 (0)	1 (6)	1 (4)	0 (0)	1 (6)
Colitis	1 (4)	0 (0)	1 (6)	1 (4)	0 (0)	1 (6)
Enterocolitis infectious	2 (8)	1 (11)	1 (6)	2 (8)	1 (11)	1 (6)
Febrile neutropenia	12 (48)	4 (44)	8 (50)	12 (48)	4 (44)	8 (50)
Fever	2 (8)	2 (22)	0 (0)	2 (8)	2 (22)	0 (0)
Gastritis	1 (4)	0 (0)	1 (6)	0 (0)	0 (0)	0 (0)
Lung infection	13 (52)	6 (67)	7 (44)	10 (40)	6 (67)	4 (25)
Mucositis oral	1 (4)	0 (0)	1 (6)	1 (4)	0 (0)	1 (6)
Salivary gland infection	1 (4)	0 (0)	1 (6)	0 (0)	0 (0)	0 (0)
Sepsis	5 (20)	3 (33)	2 (13)	5 (20)	3 (33)	2 (13)
Bleeding						
Intracranial hemmorhage	1 (4)	1 (11)	0 (0)	1 (4)	1 (11)	0 (0)
Upper gastrointestinal hemmorhage	1 (4)	1 (11)	0 (0)	1 (4)	1 (11)	0 (0)
Vitreous hemmorhage	1 (4)	1 (11)	0 (0)	1 (4)	1 (11)	0 (0)
Neurologic						
Delirium	1 (4)	1 (11)	0 (0)	1 (4)	1 (11)	0 (0)
Gait disturbance	1 (4)	0 (0)	1 (6)	1 (4)	0 (0)	1 (6)
Peripheral sensory neuropathy	1 (4)	1 (11)	0 (0)	1 (4)	1 (11)	0 (0)
Transient ischemic attack	1 (4)	1 (11)	0 (0)	0 (0)	0 (0)	0 (0)
Others						
Acute kidney injury	3 (12)	1 (11)	2 (13)	3 (12)	1 (11)	2 (13)
Abdominal pain	1 (4)	1 (11)	0 (0)	1 (4)	1 (11)	0 (0)
Atrial fibrillation	2 (8)	2 (22)	0 (0)	2 (8)	2 (22)	0 (0)
Cushingoid	1 (4)	0 (0)	1 (6)	1 (4)	0 (0)	1 (6)
Fatigue	1 (4)	0 (0)	1 (6)	1 (4)	0 (0)	1 (6)
Hypotension	1 (4)	0 (0)	1 (6)	0 (0)	0 (0)	0 (0)
Ileus	1 (4)	0 (0)	1 (6)	1 (4)	0 (0)	1 (6)
Pneumonitis	2 (8)	0 (0)	2 (13)	2 (8)	0 (0)	2 (13)
Secondary HLH^a^	1 (4)	1 (11)	0 (0)	1 (4)	1 (11)	0 (0)
Investigations^b^						
Alanine aminotransferase increased	1 (4)	0 (0)	1 (6)	1 (4)	0 (0)	1 (6)
Hyperkalemia	1 (4)	0 (0)	1 (6)	1 (4)	0 (0)	1 (6)
Hypokalemia	2 (8)	1 (11)	1 (6)	2 (8)	1 (11)	1 (6)

^a^ Hemophagocytic lymphohistiocytosis.

^b^ Excluding cytopenias.

Duration of cytopenias as well as duration of hospitalization are detailed in Table 3. Grade 3/4 thrombocytopenia persisted for > 30 days in three patients (33%) in the standard arm and six patients (38%) in the experimental arm. Grade 3/4 neutropenia resolved in less than 2 weeks for all patients. No significant difference between arms regarding time to platelet‐recovery, neutrophil‐recovery or hospital discharge was seen.

**TABLE 3:** Engraftment and hospitalization.

Interval to hematological recovery, days, median (range)	All	BeEAM	BV‐BeEAM	*p*‐value
Thrombocytes > 20 G/L	13 (4–125)	16 (7–125)	12.5 (4–86)	0.5
Thrombocytes > 50 G/L	26 (5—not reached)	24 (10—not reached)	27 (5—not reached)	1
Thrombocytes > 100 G/L	34 (5—not reached)	30 (20—not reached)	39.5 (5—not reached)	0.4
Absolute neutrophil count > 0.5 G/L	10 (8–13)	10 (9–13)	10 (8–13)	1
Absolute neutrophil count > 1.0 G/L	11 (9–20)	11 (9–14)	11 (9–20)	0.4
Duration of hospitalization	23 (20–159)	21 (20–159)	23.5 (21–56)	0.3

### 3.3 Efficacy

**FIGURE 2:**
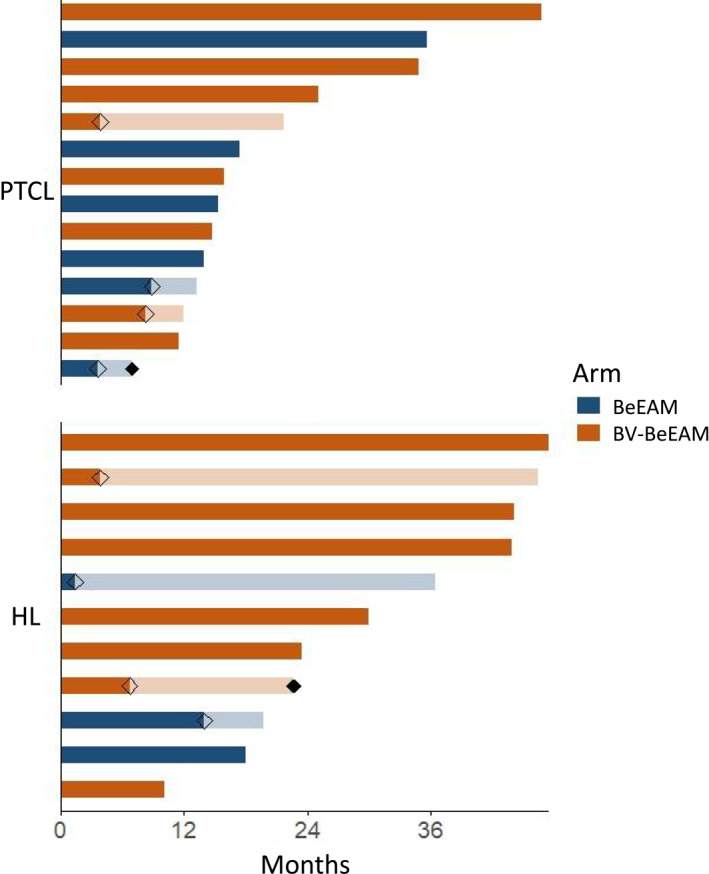
Swimmer plot depicting follow‐up of all patients in months, calculated from day of ASCT. Transparent diamonds represent progression, and transparent bars represent survival post progression. Solid diamonds represent death. Colors represent treatment arms.

Patient journeys are visualized in a swimmer plot in Figure 2. Median follow‐up for survivors was 22 months and was not significantly different between arms (18 vs. 25 m, *p* = 0.4). Five patients with HL underwent BV maintenance. For the other six, BV maintenance was omitted due to neuropathy (*n* = 1), prior pneumonitis (*n* = 2), reduced fitness after ASCT (*n* = 2) and patient preference (*n* = 1).

Relevant efficacy endpoints are detailed in Table 4. There was no significant difference between groups regarding OR‐ or CR‐rate. DFS and OS are shown in Supporting Information S1: Figure 1. No significant difference was seen for the primary endpoint, one‐year‐DFS (86% vs. 85%, *p* = 0.5). OS at 1 year was not significantly different either (89% (8/9) versus 100% (16/16), *p* = 0.4). In both cohorts, one death occurred. Both patients died due to disease progression. Per protocol, we analyzed efficacy separately for HL and TCL (Supporting Information S1: Tables 4 and 5). Neither DFS nor OS was significantly different in either HL (1‐year‐DFS: 100% vs. 83%, *p* = 0.5; 1‐year‐OS: 100% for both arms, *p* = 0.7; Supporting Information S1: Figure 2) or PTCL (1‐year‐DFS: 80% vs. 86%, *p* = 0.7; 1‐year‐OS: 100% for both arms, *p* = 0.2; Supporting Information S1: Figure 3). PFS was not significantly different for any group (Supporting Information S1: Figure 4). OS of only those achieving CR, also showed no relevant difference between groups (*p* = 0.3; Supporting Information S1: Figure 5). A post‐hoc analysis of patients without prior BV‐exposure or BV‐maintenance is detailed in Supporting Information S1: Table 6 but constrained by small numbers.

**TABLE 4:** Efficacy.

Follow‐up	All	BeEAM	BV‐BeEAM	*p*‐value
Follow‐up for survivors, months, median (range)	22 (10–48)	18 (13–36)	25 (10–48)	0.4
OR, *n* (%)	23 (92%)	8 (89%)	15 (94%)	0.7
CR, *n* (%)	20 (80%)	7 (78%)	13 (81%)	0.9
DFS at 1year, % (CI)	85 (71–100)	86 (63–100)	85 (67–100)	0.5
PFS at 1year, % (CI)	72 (56–92)	67 (42–100)	75 (57–100)	0.3
OS at 1year, % (CI)	96 (89–100)	89 (71–100)	100 (NA)	0.4
Death^a^, *n* (%)	2 (8%)	1 (11%)	1 (6%)	—

Abbreviations: CR, Complete Response; DFS, Disease Free Survival; OR, Objective Response; OS, Overall Survival; PFS, Progression Free Survival.

^a^all deaths were due to progression of disease.

## 4 Discussion

This phase II investigator‐initiated trial was designed based on the hypothesis that the addition of BV to HDCT could induce deeper and/or more durable remissions after ASCT in all CD30+ lymphoma types.

Previous studies have shown that most patients with HL can be cured, but over 10% relapse even after intensive first‐line combination chemotherapy [35]. Treating these patients using HDCT induces durable remissions in about 50 percent of cases [2, 36, 37, 38, 39]. Those relapsing after ASCT have a dismal prognosis [40]. PTCL generally have a worse prognosis than HL, and therefore typically undergo HDCT and ASCT as consolidation in first remission. They too have a much worse prognosis when relapsing after ASCT [41]. As illustrated in the introduction, BV has entered treatment algorithms of both entities, being used in first‐line, salvage and maintenance regimens. With the addition of BV to HDCT being safe in phase I of this trial [33], a randomized evaluation of the efficacy of this combination was planned to elucidate whether BV would also be useful in this therapeutic setting.

Here, the toxicity of BV‐BeEAM once again proved manageable. Median days of hospitalization were not significantly different between treatment arms, and neutrophil recovery was virtually unaffected by the addition of BV. Infectious toxicities, though frequent as expected for HDCT, were similar between arms. No treatment related deaths occurred. However, two cases of pneumonitis occurred in the experimental arm. One occurred at day 35 after application of BV, and the other occurred about 3 months after BV‐application, with no further doses being applied in the meantime. Both were grade 3, were treated with a steroid pulse and resolved within less than 2 weeks. Neither necessitated ICU‐admission or mechanical ventilation. Even though both cases resolved completely, pneumonitis is a known risk of BV, with 5% of patients in the experimental arm of the AETHERA trial having some pulmonary toxicity [3, 42]. Thus, a potential increase in pulmonary toxicity should be considered when adding BV to HDCT.

While the control arm and experimental arm were largely homogenous, a slight bias toward lesser BV‐exposure and higher CR rate at diagnosis in the standard arm should—if relevant at all at the time of HDCT—give a marginal benefit to the experimental arm. Despite that, the efficacy of BV‐BeEAM was no better than BeEAM alone. AS HDCT is expected to lead to better outcomes in HL than in PTCL, we analyzed both disease entities separately. Again, no benefit was observed. Another crucial disease characteristic is the response to salvage chemotherapy prior to ASCT. For HL, one study reported a 10‐year‐OS of 66% versus 17% in responders and non‐responders respectively [43], while in PTCL, one study reported a 3‐year‐OS of 89% versus 65% in responders versus non‐responders [44]. However, when analyzing only those who achieved a CR after HDCT in our study, once again there was no meaningful difference between the two treatments.

Multiple reasons should be considered for the disappointing efficacy of BV‐BeEAM. One is the frequent exposure to BV in other settings. In HL, BrECADD has recently shown superiority to eBEACOPP [16]. In addition, on long‐term follow‐up BV‐maintenance for HL continues to prove beneficial [3]. Between conception and completion of this study, BV has also entered the first‐line in PTCL, as the 5‐year follow‐up of ECHELON‐2 has shown an OS‐benefit of 70.1% versus 61.0% when using BV‐CHP instead of CHOP [30]. Unsurprisingly, 11 of our patients had prior exposure to BV(1 HL‐patient: prior BrECADD, 10/14 PTCL‐patients: prior BV‐CHP), and 5 (all HL) underwent BV‐maintenance. As our protocol only included one dose of BV, any effect of the ADC may have been hidden behind the greater effect of the multiple doses applied in other settings or may have been too small to become measurable after just one dose. This hypothesis could not be empirically tested unfortunately, as only two patients in the control arm who both neither had a DFS‐ or OS‐event were not exposed to BV outside of the study, thus precluding any post‐hoc comparison of those who only had BV in conjunction with BeEAM. As finding more patients without BV‐exposure would become increasingly more difficult due to the increased importance of BV outlined above, we believe that continuing the study as planned would likely not have answered this question either. However, the fact that BV‐pretreatment and maintenance were allowed constitute a methodical strength of the study, as only this way the relevance of BV‐BeEAM in the current therapeutic landscape can be elucidated. One potential limitation of this study is the premature termination, which could theoretically contribute to an existing effect of the experimental arm being missed (increased probability for type II error) [45]. Nonetheless, the clear conclusions of the futility analysis coupled with the observed increase in pulmonary toxicity make it clear that the decision to not expose more patients to an investigational therapy unlikely to prove beneficial was correct.

In conclusion, the addition of BV to BeEAM for HDCT in CD30+ lymphoma planned to undergo ASCT may increase the risk of pulmonary toxicity without improving efficacy. BV should therefore be reserved for other time‐points of the treatment journey.

## Author Contributions

C.R.: analyzed data, designed the figures, and wrote the paper, U.B., M.R., C.V., A.K., and M.D.: analyzed data and wrote the paper, Y.B.: provided reference pathology, T.P.: designed research, analyzed data, and wrote the paper. All authors were involved in drafting the manuscript, and all authors read and approved the final version. All authors have read and agreed to the published version of the manuscript.

## Conflicts of Interest

C.R. received travel grants from IME, Kite‐Gilead, JAZZ Pharmaceuticals and Servier. He participated in advisory boards for BeiGene and Takeda. None of the advisory boards directly related to the work presented here. T.P. received research grants from Millenium Pharmaceuticals Inc. and Takeda. All other authors declare no conflicts of interest.

## Peer Review

The peer review history for this article is available at https://www.webofscience.com/api/gateway/wos/peer-review/10.1002/hon.70143.

## Supporting information


Supporting Information S1

## Data Availability

Deidentified individual participant data are available from the corresponding author upon reasonable request.
