# Supplementary material for: Randomized Phase II Study of Brentuximab‐Vedotin With High‐Dose Chemotherapy in CD30 Positive Lymphoma
Source: Hematol Oncol. 2025 Oct 21;43(6):e70143. doi: 10.1002/hon.70143 (PMC12539077; doi:10.1002/hon.70143)
Supplement: Supplementary file 1 — Supporting Information S1 [file HON-43-e70143-s001.docx]

### Supplemental Tables

#### Supplemental Table 1: Eligibility Criteria.

| **Inclusion criteria** | **Exclusion criteria** |
| --- | --- |
| CD30+ malignant lymphoma in 1^st^ or 2^nd^ remission or 2^nd^ chemo-sensitive relapse | Relevant co-existing disease excluding a treatment according to protocol |
| HDCT/ASCT planned | Patient not fit for ASCT |
| Age 18-75 | Lack of patient cooperation to allow study treatment as outlined in this protocol |
| Written informed consent | Pregnant or lactating female patients |
| Negative pregnancy test within 14 days prior to registration (women of childbearing potential) | Concurrent malignant disease (except basal cell / squamous cell carcinoma of the skin, early-stage cervix carcinoma, or early-stage prostate cancer) |
| Agreement to use effective contraception from registration until 12 months after completion of treatment | Previous treatment for other malignancies (not listed above) terminated less than 24 months before registration, or evidence of active disease since then |
| Platelets ≥ 75x10^9^/L unless there is known marrow involvement of the disease | Major coagulopathy or bleeding disorder |
| Absolute neutrophils ≥ 1,5x10^9^/L, unless there is known marrow involvement of the disease | Major surgery less than 30 days before start of treatment |
| Total bilirubin < 1.5 x ULN unless the elevation is known to be due to Gilbert syndrome | Known history of any of the following cardiovascular conditions:  - Myocardial infarction in the last 2 years   - NYHA Class III or IV heart failure   - Current uncontrolled cardiovascular conditions  - Recent evidence (6 months before 1^st^ dose of study drug) of LVEF <50% |
| ALT or AST < 3 x ULN or AST and ALT < 5 x ULN, if the malignancy involves the liver | Symptomatic neurologic disease compromising normal activities of daily living or requiring medications |
| Serum creatinine < 2.0 mg/dL and/or  (calculated) creatinine clearance > 40 mL/min | Any sensory or motor peripheral neuropathy greater than or equal to grade 2 |
| Hemoglobin ≥ 8g/dL | Any prior treatment not terminated for at least 5 half-lives of last dose of that treatment before inclusion |
|  | Known hypersensitivity to any excipient contained in the drug formulation of Brentuximab Vedotin |
|  | Acute uncontrolled infection |

#### Supplemental Table 2: Baseline characteristics of patients with Hodgkin Lymphoma

| **Demographic Characteristics** | **All** | **BeEAM** | **BV-BeEAM** | **p-value** |
| --- | --- | --- | --- | --- |
| **n** | 11 | 3 | 8 | - |
| **Age at ASCT, y, median (range)** | 56.0 (19.6-73.0) | 56.0 (39.8-60.3) | 57.2 (19.6-73.0) | 0.8 |
| **Gender, female, n (%)** | 2 (18%) | 1 (33.3%) | 1 (12.5%) | 0.5 |
| **HL subtypes, n (%)** | | | | |
| **cHL** | 9 (82%) | 3 (100%) | 6 (75%) | - |
| **Mixed-type HL** | 2 (18%) | 0 (0%) | 2 (25%) | - |
| **CD30+ lymphocytes at diagnosis, %, median (range)** | 1.0 (0.3-1.0) | 1.0 (1.0-1.0) | 1.0 (0.3-1.0) | 0.2 |
| **Stage (Ann-Arbor), n (%):** | | | | |
| **I** | 1 (9%) | 0 (0%) | 1 (13%) | **0.03** |
| **II** | 2 (18%) | 1 (33.3%) | 1 (13%) |  |
| **III** | 4 (36%) | 0 (0%) | 4 (50%) |  |
| **IV** | 4 (36%) | 2 (66.7%) | 2 (25%) |  |
| **ECOG-PS:** | | | | |
| **0** | 10 (91%) | 2 (67%) | 8 (100%) | 0.1 |
| **1** | 1 (9%) | 1 (33%) | 0 (0%) |  |
| **Extranodal involvement, n (%) **** | 6 (55%) | 2 (67%) | 4 (50%) | 1 |
| **Extranodal sites, n** | | | | |
| Bone marrow | 3 | 2 | 1 | - |
| Intestine | 1 | 0 | 1 | - |
| Kidney | 1 | 0 | 1 | - |
| Liver | 2 | 0 | 2 | - |
| Mediastinum | 1 | 0 | 1 | - |
| **Previous therapies:** | | | | |
| **Prior lines of chemotherapy, median (range)** | 2 (2-4) | 3 (2-3) | 2 (2-4) | 0.6 |
| **Regimens used, n** | | | | |
| Prior ABVD | 3 | 0 | 3 | - |
| Prior AVD | 1 | 1 | 0 | - |
| Prior BEACOPP | 5 | 2 | 3 | - |
| Prior BrECADD | 1 | 0 | 1 | - |
| Prior DHAOx | 3 | 1 | 2 | - |
| Prior DHAP | 8 | 3 | 5 | - |
| Prior ICE | 1 | 0 | 1 | - |
| Prior ICE + Pembrolizumab | 1 | 0 | 1 | - |
| Prior IGEV | 1 | 0 | 1 | - |
| Prior O-CHOP* | 1 | 1 | 0 | - |
| Prior Pembrolizumab | 1 | 0 | 1 | - |
| Prior Stanford V | 1 | 0 | 1 | - |
| **Prior radiotherapy, n (%)** | 2 (18%) | 1 (33%) | 1 (13%) | 0.5 |
| **Prior exposure to BV** | 1 (9%) | 0 (0%) | 0 (0%) | 1 |
| **BV_maintenance, n (%)** | 5 (46%) | 2 (67%) | 3 (38%) | 0.5 |
| **CR at randomisation, n (%)** | 6 (55%) | 1 (33%) | 5 (63%) | 0.5 |
| **Time from diagnosis to ASCT, months, median (range)** | 16.9 (6.8-91.4) | 16.9 (11.2-20.9) | 33.9 (6.8-91.4) | 0.7 |
| **Remission status before ASCT, n (%):** | | | | |
| **CR** | 6 (55%) | 1 (33.3%) | 5 (63%) | 0.3 |
| **PR** | 3 (27%) | 2 (66.7%) | 1 (13%) |  |
| **SD** | 1 (9%) | 0 (0%) | 1 (13%) |  |
| **PD** | 1 (9%) | 0 (0%) | 1 (13%) |  |

ASCT = autologous stem cell transplant; ABVD = doxorubicin, bleomycin, vinblastine, and dacarbazine; AVD = doxorubicin, vinblastine, and dacarbazine; BEACOPP = bleomycin, etoposide, doxorubicin, cyclophosphamide, vincristine, procarbazine, and prednisone; BrECADD = brentuximab vedotin, etoposide, cyclophosphamide, adriamycin, dacarbazin, and dexamethasone; BV = Bentuximab-Vedotin; cHL = classical Hodgkin Lymphoma; CR = complete response; DHAP = dexamethasone, high-dose cytarabine, and cisplatin; DHAOx = dexamethasone, high-dose cytarabine, and oxaliplatin; ECOG-PS = eastern cooperative group performance status; HL = Hodgkin Lymphoma; ICE = ifosfamide, cisplatin and etoposide; IGEV = ifosfamide, gemcitabine, vinorelbine, and prednisone; PD = progressive disease; PR = partial response; SD = stable disease; Stanford V = mechlorethamine, doxorubicin, vinblastine , vincristine , bleomycin, etoposide, and prednisone; * Received for FL, which transformed to cHL

#### Supplemental Table 3: Baseline characteristics of patients with T-Cell Lymphoma

| **Demographic Characteristics** | **All** | **BeEAM** | **BV-BeEAM** | **p-value** |
| --- | --- | --- | --- | --- |
| **n** | 14 | 6 | 8 | - |
| **Age at ASCT, y, median (range)** | 61.1 (36.2-71.6) | 63.8 (49.4-71.6) | 59.8 (36.2-71.6) | 0.8 |
| **Gender, female, n (%)** | 5 (36%) | 1 (17%) | 4 (50%) | 0.3 |
| **TCL Subtypes** | | | | |
| **AITL** | 5 | 0 | 5 | - |
| **ALCL** | 2 | 2 | 0 | - |
| **EATL** | 1 | 1 | 0 | - |
| **PTCL NOS** | 4 | 2 | 2 | - |
| **TFHL** | 2 | 1 | 1 | - |
| **CD30+ lymphocytes at diagnosis, %, median (range)** | 0.4 (0.05-1.0) | 0.75 (0.40-1.0) | 0.13 (0.05-0.9) | **0.04** |
| **Stage (Ann-Arbor), n (%):** | | | | |
| **I** | 0 (0%) | 0 (0%) | 0 (0%) | **0.04** |
| **II** | 1 (7%) | 1 (17%) | 0 (0%) |  |
| **III** | 3 (21%) | 1 (17%) | 2 (25%) |  |
| **IV** | 10 (71%) | 4 (67%) | 6 (75%) |  |
| **IPI, n (%):** | | | | |
| **0** | 0 (0%) | 0 (0%) | 0 (0%) | **0.02** |
| **1** | 3 (21%) | 1 (17%) | 2 (25%) |  |
| **2** | 5 (36%) | 2 (33%) | 3 (38%) |  |
| **3** | 5 (36%) | 2 (33%) | 3 (38%) |  |
| **4** | 0 (0%) | 0 (0%) | 0 (0%) |  |
| **5** | 1 (7%) | 1 (17%) | 0 (0%) |  |
| **ECOG-PS:** | | | | |
| **0** | 13 (93%) | 6 (100%) | 7 (88%) | 0.1 |
| **1** | 1 (7%) | 0 (0%) | 1 (12%) |  |
| **Extranodal involvement, n (%)** | 7 (50%) | 3 (50%) | 4 (50%) | 1.0 |
| **Extranodal sites, n** | | | | |
| Bone marrow | 1 | 0 | 1 | - |
| Gastric | 1 | 0 | 1 | - |
| Kidney | 1 | 0 | 1 | - |
| Lungs | 3 | 2 | 1 | - |
| Mesenterium | 1 | 1 | 0 | - |
| Pharynx | 1 | 1 | 0 | - |
| Skin | 1 | 0 | 1 | - |
| **Previous therapies:** | | | | |
| **Prior lines of chemotherapy, median (range)** | 2.0 (1-4) | 1.5 (1-2) | 2.0 (1-4) | 0.2 |
| **Regimens used, n** | | | | |
| Prior BV-CHP | 10 | 5 | 5 | - |
| Prior CHOEP | 3 | 0 | 3 | - |
| Prioc CHOP | 9 | 4 | 5 | - |
| Prior DHAP | 1 | 0 | 1 | - |
| Prior gemcitabine and carboplatin | 2 | 0 | 2 | - |
| Prior gemcitabine and oxaliplatin | 1 | 0 | 1 | - |
| **Prior radiotherapy, n (%)** | 1 (7%) | 0 (0%) | 1 (12%) | 0.3 |
| **Prior exposure to BV** | 10 (71%) | 5 (83%) | 5 (63%) | 0.6 |
| **Time from diagnosis to ASCT, months, median (range)** | 6.3 (5.6-199.3) | 6.5 (5.7-199.3) | 6.1 (5.6-17.6) | 0.3 |
| **Remission status before ASCT, n (%):** | | | | |
| **CR** | 11 (79%) | 4 (67%) | 7 (88%) | 0.1 |
| **PR** | 3 (21%) | 2 (33%) | 1 (13%) |  |
| **SD** | 0 (0%) | 0 (0%) | 0 (0%) |  |
| **PD** | 0 (0%) | 0 (0%) | 0 (0%) |  |

AITL = angioimmunoblastic T-cell lymphoma; ALCL = anaplastic large-cell lymphoma; ASCT = autologous stem cell transplant; BV = Bentuximab-Vedotin; BV-CHP = brentuximab vedotin, cyclophosphamide, doxorubicin, and prednisone; CHOEP = cyclophosphamide, doxorubicin, etoposide, vincristine, and prednisone; CHOP = cyclophosphamide, doxorubicin, vincristine, and prednisone; CR = complete response; DHAP = dexamethasone, high-dose cytarabine, and cisplatin; EATL = enteropathy associated T-cell lymphoma; ECOG-PS = eastern cooperative group performance status; IPI = international prognostic index; PD = progressive disease; PR = partial response; PTCL NOS = peripheral T-cell lymphoma not otherwise specified; SD = stable disease; TCL = T-cell lymphoma; TFHL = follicular helper T-cell lymphoma.

#### Supplemental Table 4: Efficacy in patients with HL

| **Follow-up** | **All** | **BeEAM** | **BV-BeEAM** |  |
| --- | --- | --- | --- | --- |
| Follow-up for survivors, months, median (range) | 33 (10 - 48) | 20 (18 - 36) | 44 (10 - 48) | 0.3 |
| OR, n (%) | 9 (82%) | 2 (67%) | 7 (88%) | 0.5 |
| CR, n (%) | 8 (73%) | 2 (67%) | 6 (75%) | 0.9 |
| DFS at 1y, % (CI) | 88 (67 - 100) | 100 (NA) | 83 (58 - 100) | 0.5 |
| PFS at 1y, % (CI) | 72 (51 - 100) | 67 (30 - 100) | 75 (50 - 100) | 0.2 |
| OS at 1y, % (CI) | 100 (NA) | 100 (NA) | 100 (NA) | 0.7 |
| **Death, n (%)** | 1 (9) | 0 (0) | 1 (13) | - |

CR = Complete Response, DFS = Disease Free Survival, OR = Objective Response, OS = Overall Survival, PFS = Progression Free Survival; *all deaths were due to progression of disease

#### Supplemental Table 5: Efficacy in patients with PTCL

| **Follow-up** | **All** | **BeEAM** | **BV-BeEAM** |  |
| --- | --- | --- | --- | --- |
| Follow-up for survivors, months, median (range) | 16 (12 - 47) | 15 (13 - 36) | 19 (12 - 47) | 0.8 |
| OR, n (%) | 14 (100) | 6 (100) | 8 (100) | - |
| CR, n (%) | 12 (86) | 5 (83) | 7 (88) | 0.9 |
| DFS at 1y, % (CI) | 83 (11 - 65) | 80 (18 - 52) | 86 (12 - 63) | 0.7 |
| PFS at 1y, % (CI) | 71 (51 - 100) | 67 (38 - 100) | 75 (50 - 100) | 0.7 |
| OS at 1y, % (CI) | 93 (80 - 100) | 83 (58 - 100) | 100 (NA) | 0.2 |
| **Death, n (%)** | 1 (7) | 1 (17) | 0 (0) | - |

CR = Complete Response, DFS = Disease Free Survival, OR = Objective Response, OS = Overall Survival, PFS = Progression Free Survival; *all deaths were due to progression of disease

#### Supplemental Table 6: Efficacy in patients without prior BV-Exposure or BV-maintenance

|  | **All (n = 9)** | | **HL (n=5)** | | **PTCL (n = 4)** | |
| --- | --- | --- | --- | --- | --- | --- |
| **Follow-up** | **BeEAM** | **BV-BeEAM** | **BeEAM** | **BV-BeEAM** | **BeEAM** | **BV-BeEAM** |
| Follow-up for survivors, months, median (range) | 17 (15 - 18) | 28 (22 - 46) | 18 (NA) | 30 (23 - 46) | 15 (NA) | 25 (22 - 35) |
| OR, n (%) | 2 (100) | 6 (86) | 1 (100) | 3 (75) | 1 (100) | 3 (100) |
| CR, n (%) | 2 (100) | 5 (71) | 1 (100) | 3 (75) | 1 (100) | 2 (67) |
| DFS at 1y, % (CI) | 100 (NA) | 80 (52 - 100) | 100 (NA) | 67 (30 - 100) | 100 (NA) | 100 (NA) |
| PFS at 1y, % (CI) | 100 (NA) | 57 (30 - 100) | 100 (NA) | 50 (19 - 100) | 100 (NA) | 67 (30 - 100) |
| OS at 1y, % (CI) | 100 (NA) | 100 (NA) | 100 (NA) | 100 (NA) | 100 (NA) | 100 (NA) |
| Death, n (%) | 2 (100) | 1 (14) | 0 (0) | 1 (25) | 0 (0) | 0 (0) |

CR = Complete Response, DFS = Disease Free Survival, OR = Objective Response, OS = Overall Survival, PFS = Progression Free Survival; *all deaths were due to progression of disease

### Supplemental Figures

#### Supplemental Figure 1: DFS and OS of all patients


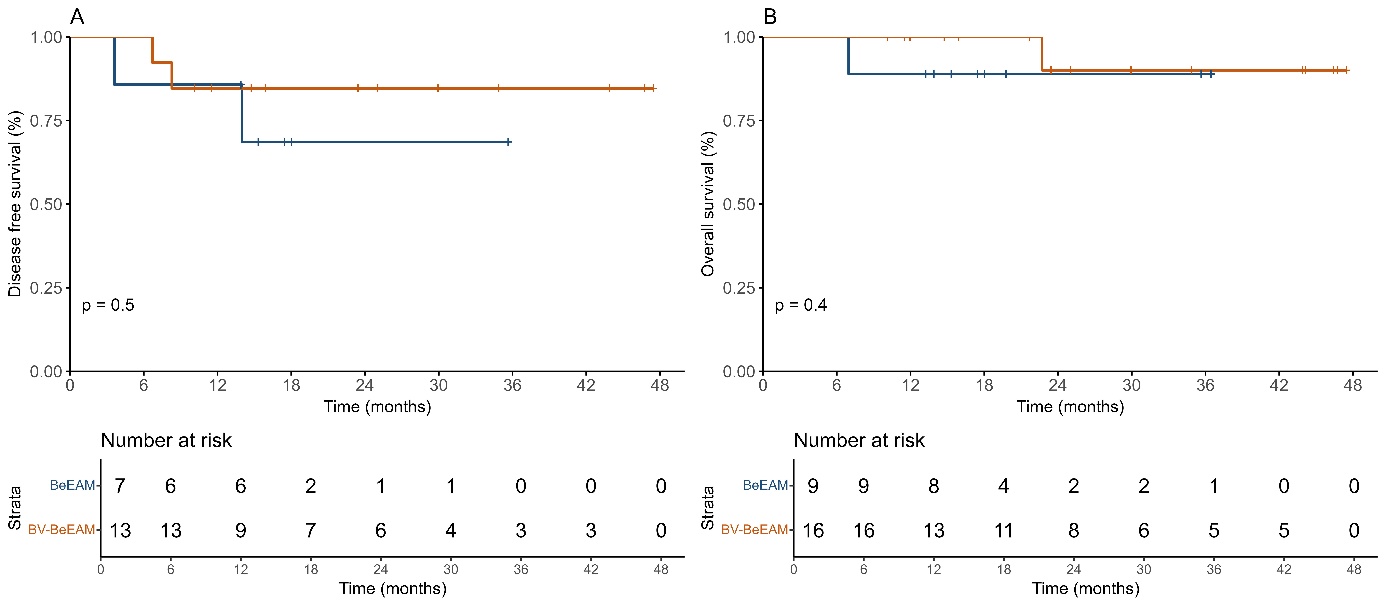


#### Supplemental Figure 2: DFS and OS of patients with HL


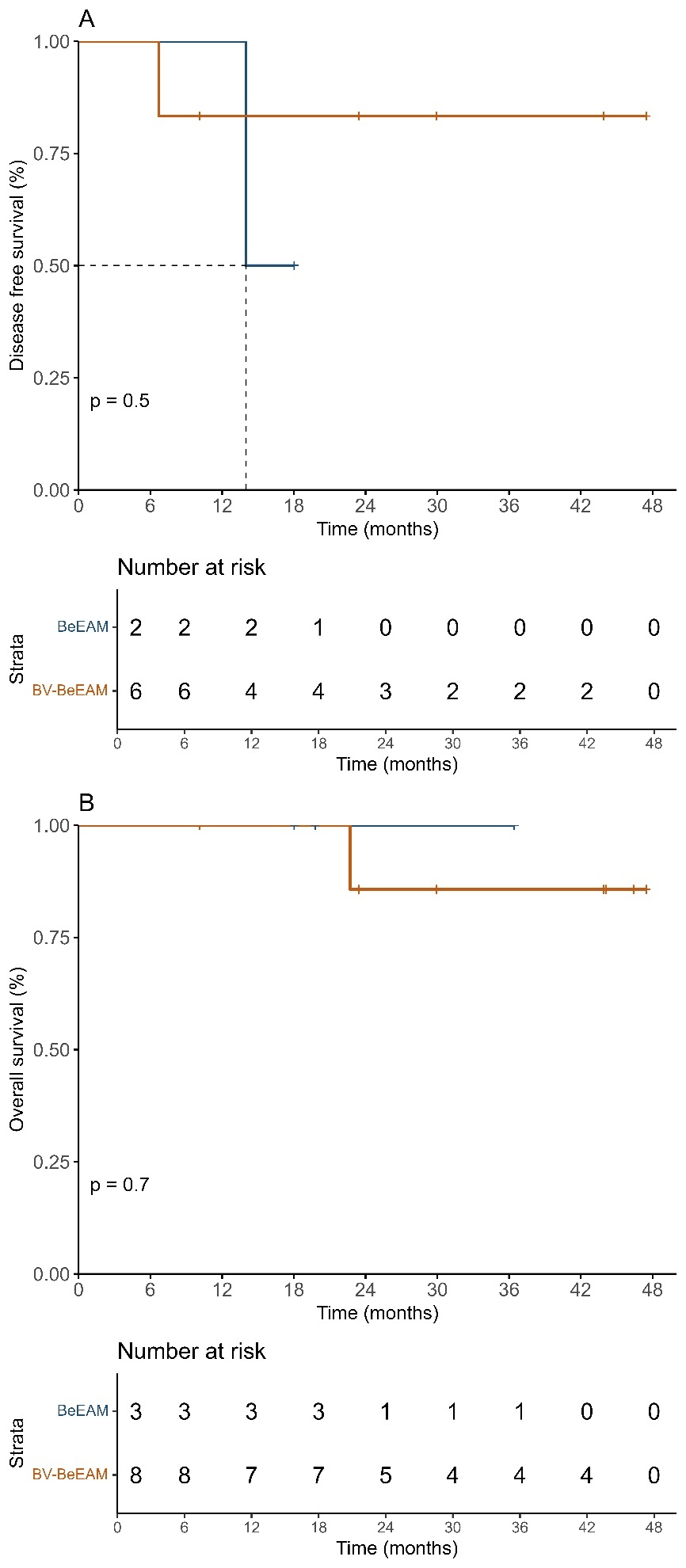


#### Supplemental Figure 3: DFS and OS of patients with PTCL


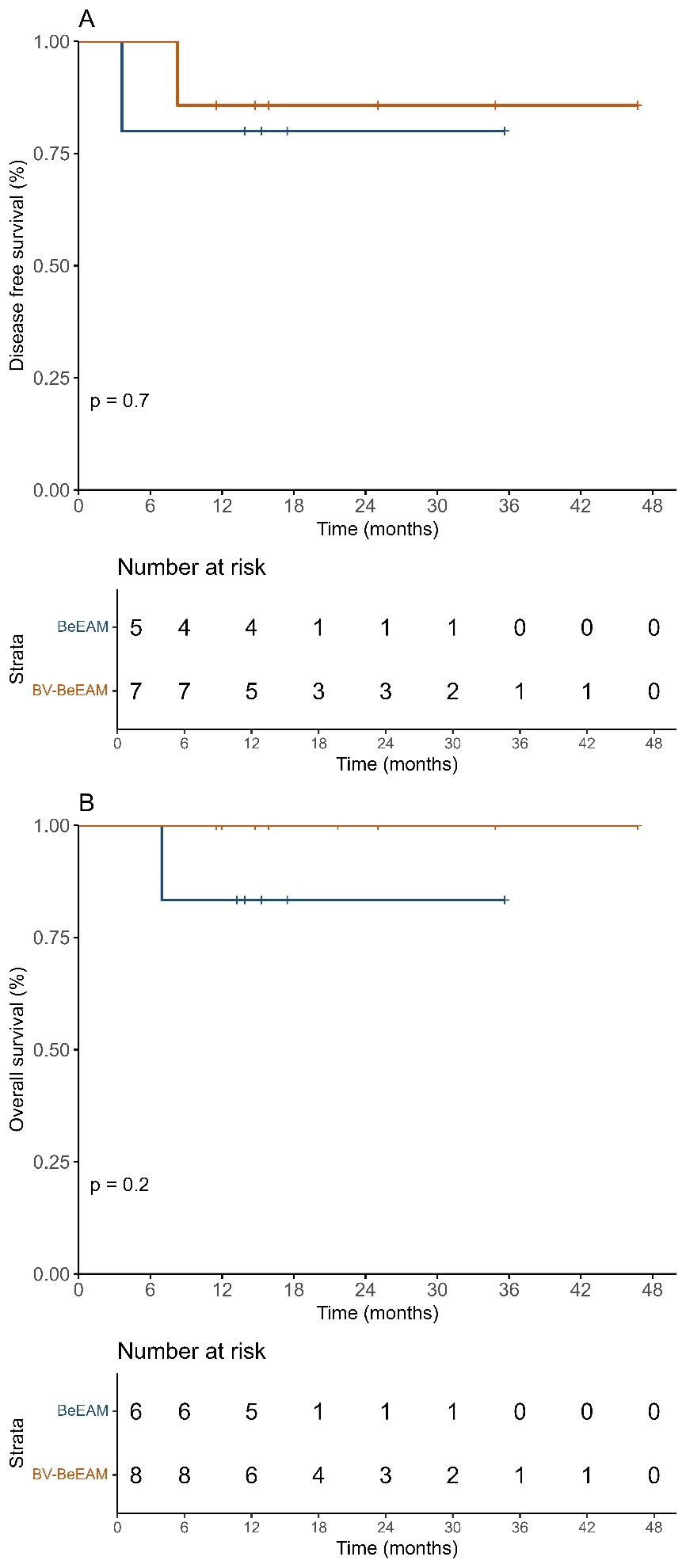


#### Supplemental Figure 4: PFS of all patients, and disease subgroups


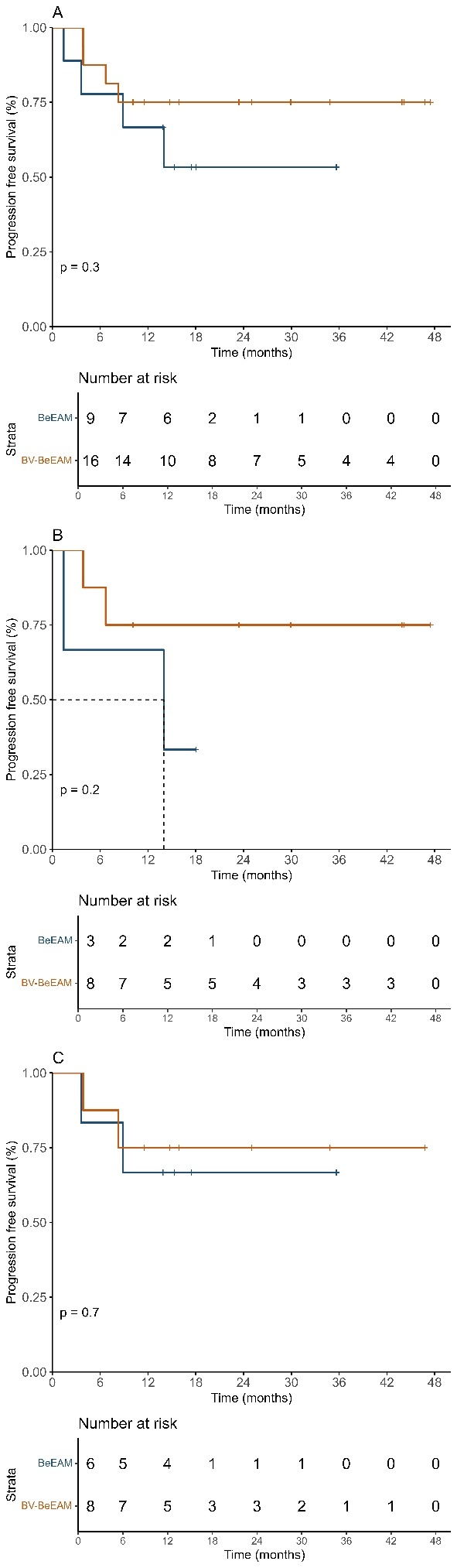


#### Supplemental Figure 5: OS of patients in CR after ASCT


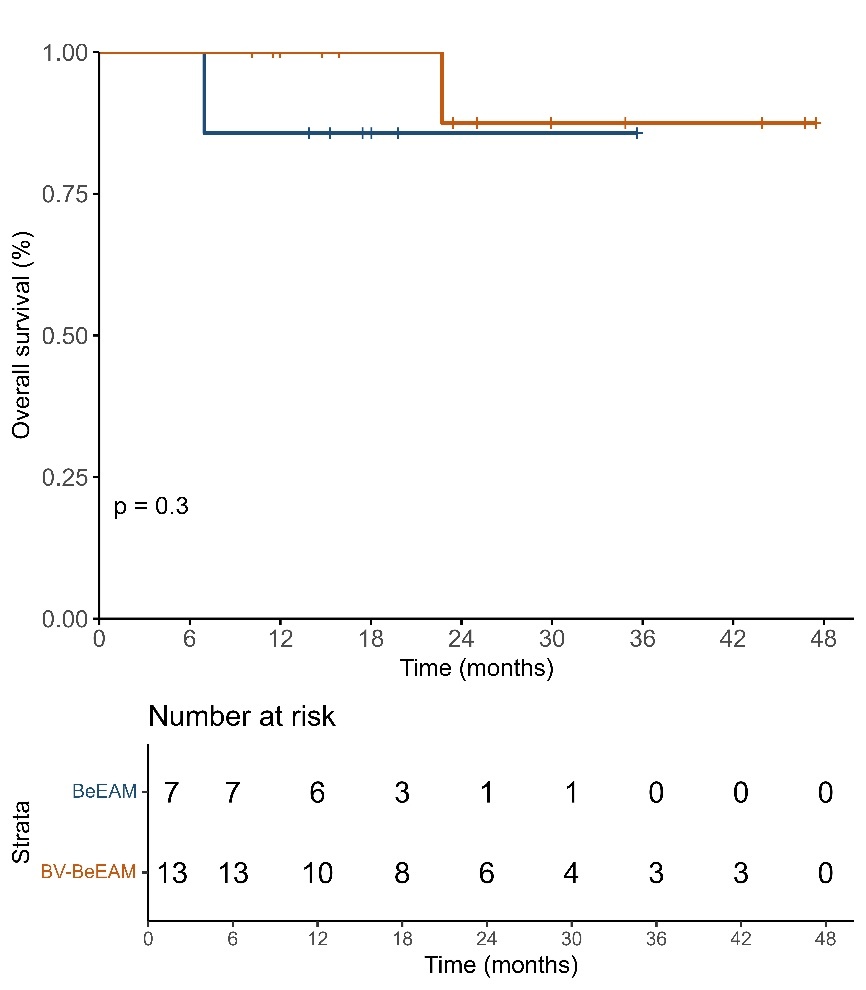


### Supplemental Figure Legends

Supplemental Figure 1: Kaplan-Meier estimation of DFS (Panel A) and OS (Panel B) of all 25 patients. Treatment arms are compared using the log-rank test. Tick marks indicate censoring events.

Supplemental Figure 2: Kaplan-Meier estimation of DFS (Panel A) and OS (Panel B) of patients with HL. Treatment arms are compared using the log-rank test. Tick marks indicate censoring events. The dashed lines represent the median survival probability.

Supplemental Figure 3: Kaplan-Meier estimation of DFS (Panel A) and OS (Panel B) of patients with PTCL. Treatment arms are compared using the log-rank test. Tick marks indicate censoring events.

Supplemental Figure 4: Kaplan-Meier estimation of PFS of all patients (Panel A), and disease subgroups (HL: Panel B; PTCL: Panel C). Treatment arms are compared using the log-rank test. Tick marks indicate censoring events. The dashed lines represent the median survival probability.

Supplemental Figure 5: Kaplan-Meier estimation of OS of all patients who reached CR after ASCT. Treatment arms are compared using the log-rank test. Tick marks indicate censoring events.
